# Knowledge, Attitudes and Practices of Adults in the United Arab Emirates Regarding *Helicobacter pylori* induced Gastric Ulcers and Cancers

**DOI:** 10.31557/APJCP.2021.22.5.1645

**Published:** 2021-05

**Authors:** Abdullah Imadeddin Malek, Muzan Abdelbagi, Lian Odeh, Atheer Tariq Alotaibi, Mohamed Husain Alfardan, Hiba Jawdat Barqawi

**Affiliations:** *Department of Clinical Sciences, University of Sharjah, Sharjah, United Arab Emirates. *

**Keywords:** Helicobacter pylori, gastric ulcers, gastric cancers, public health, community health

## Abstract

**Objective:**

The aim of this study is to assess the current level of knowledge, as well as the attitudes and practices (KAP) of the adult population in Sharjah, UAE with regards to *H. pylori *induced gastric ulcers and gastric cancers.

**Methods:**

A cross sectional study of 500 participants was conducted in public venues in Sharjah, UAE through the distribution of a self-administered questionnaire English and Arabic speaking residents aged 18 years and above of both sexes were invited to participate in this study via convenience sampling. Responses were collected and analyzed using SPSS.

**Results:**

General knowledge about *H. pylori* was poor, only 24.6% had heard of *H. pylori*. 61% of the participants did not know the link between *H. pylori* and gastric cancer. Only 3% of the participants associated psychological stress with gastric ulcer development. Females had higher knowledge scores (p = 0.008*). Participants with a medical background typically had higher knowledge scores than their peers in other fields of work (p < 0.0001*). Participants’ attitudes towards *H. pylori* were suboptimal with only 33% willing to seek medical help If they get symptoms. Majority of participants with an approximate of 84% showed an overall average to excellent practices towards *H. pylori*.

**Conclusion:**

General awareness about *H. pylori* induced gastric ulcers and cancers is poor. The results of this study can be a starting point to devise new education programs and campaigns that raise awareness of this health issue which could be easily avoided with prevention, early detection, and intervention.

## Introduction

One million deaths worldwide can be annually attributed to gastric ulcers and gastric cancer, specifically in low- to middle-income populations (Ferlay et al., 2015; Plummer et al., 2016). *Helicobacter pylori* (*H. pylori*) infection and non-steroidal anti-inflammatory drugs (NSAIDs) are the most common risk factors for the development of gastric ulcers (Kuna et al., 2019). *H. pylori* is a gram-negative bacillus that can survive in the gastric mucosa and contribute to significant inflammation and tissue damage due to a multitude of virulence factors. Globally, *H. pylori* is believed to infect more than half of the world’s population with high geographical variability (Hooi et al., 2017). *H. pylori* prevalence of 74-78% has been reported in the asymptomatic population of the UAE (Eshraghian, 2014). To the best of our knowledge, no similar data that assess knowledge, attitudes and prevalence have been documented in the UAE. Since most patients remain asymptomatic for a long period of time, long-term colonization of the gastric mucosa is thought to be an important factor in the future development of several diseases such as chronic gastritis, peptic ulcer disease, gastric cancer, and mucosa-associated lymphoid tissue (MALT) lymphoma (Cover and Blaser, 2009; Muhammad et al., 2013). The progression of *H. pylori* to gastric cancer is a complex multistep process that may take decades to develop from chronic gastritis to atrophic gastritis to intestinal dysplasia and eventually, cancer. This latency period offers a window of opportunity for early detection and eradication of *H. pylori* prior to the manifestation of any complications. Three cohort studies led to the classification of *H. pylori* as a class I carcinogen by the World Health Organization (WHO) (Moss, 2017). A meta-analysis conducted by Plummer et. Al. reported that 89.0% of non-cardia gastric cancer was attributable to *H. pylori* (Plummer et al., 2016). Despite the overall decline in the incidence and mortality rates of gastric cancer around the world, it remains a major health concern. Gastric cancer is the fifth most common cancer in the world (“Cancer today”, 2020) and is responsible for 783,000 deaths globally, making it the third most common cause of death due to cancer (Bray et al., 2018). Eradication of *H. pylori* involves a standard triple therapy that consists of a proton pump inhibitor, clarithromycin and either metronidazole or amoxicillin. Successful eradication and treatment of *H. pylori* leads to regression of acute and chronic inflammation (Li et al., 2017), healing of gastric ulcers (Ford et al., 2016; Leodolter et al., 2001) and prevention of gastric cancer (Wong et al., 2004). However, the success rates of the standard therapy have become suboptimal in many parts of the world falling below an acceptable level of 80% (Graham et al., 2007). This could be attributed to antibiotic resistance, patient noncompliance and several host or bacterial factors that may hinder the efficacy of eradication (Kim et al., 2015). There is a generalized consensus regarding poor knowledge and awareness of the general population about the impending effects of *H. pylori* and its possible complications (Chen et al., 2005) (Oh et al., 2009) (Shin et al., 2013) (Wynne et al., 2013). Attitudes related to *H. pylori* infection were unexpected. Some studies have assessed the perception of self-risk of their participants. Most have viewed their risk of contracting *H. pylori* infection and developing stomach cancer as “similar to” or lower than their peers of the same gender and age (Oh et al., 2009) (Wynne et al., 2013) while others did not believe they would be infected with *H. pylori* although its prevalence was around 40% in that environment (Xia et al., 2012). Commonly, good habits are being practiced and have been associated with less risks of *H. pylori* infection (Lee et al. 2012). These studies found the inadequate number of publications regarding *H. pylori* surprising given the fact that gastric cancer attributed to *H. pylori* infection can be prevented through strategies like screening and early treatment. This study aims to assess the current level of KAP of the residents of Sharjah, UAE with regards to *H. pylori* gastric ulcers with the intent to promote early detection and eradication and raise awareness about the dangers of untreated *H. pylori* infection.

## Materials and Methods

*Research design*

A descriptive, cross-sectional research design was implemented to assess the levels of KAP of the population in Sharjah, UAE, with regards to gastric ulcers and *H. pylori*.

*Questionnaire design*

Considering the lack of current tools evaluating *H. pylori* KAPs, particularly among high-risk populations, a tool was developed by the authors using the World Health Organization Guide to Developing Knowledge, Attitude, and Practice Surveys (WHO, 2008). Additionally, literature and international studies were used for further guidance on how to structure the questionnaire (Canbaz, Sunter, Peksen and Leblebicioglu, 2005) (Ahmed, Salih, Jafri, Ali Shah and Hamid, 2009) (Abebaw et al., 2014) (Tsongo et al., 2015) (Driscoll et al., 2017) (Cano-Contreras et al., 2018) (Wu et al., 2020). The questionnaire was developed in English then translated to Arabic.

The questionnaire included 18 questions and consisted of 4 sections: demographics (8), knowledge (6), attitudes (2), and practices (2) of the population in Sharjah towards *H. Pylori*. Demographics questions collected data regarding the gender, age, nationality, educational level, and field of work of the participants. Participants were also asked about any current symptomatology or any previous health conditions. Knowledge was assessed using multiple choice questions that aimed to gauge the level of knowledge the participants had with regards to gastric ulcers generally and *H. pylori* specifically. Attitudes regarding the prevention of gastric ulcers and avoiding infection by *H. pylori* were also assessed using multiple choice questions and a 3-point Likert scale. Practices included multiple choice questions about practices the participants engaged in on a regular basis. 

*Sampling and pilot study*

A minimum sample size of 385 was calculated based on 5% marginal error and 50% prevalence using the following formula: where n = sample size, p = expected prevalence, and SE = sampling error. . A pilot study was initially conducted with 25 volunteers. After modification and review, based on feedback from the pilot study, the questionnaire was approved by the Ethics Committee in the University of Sharjah prior to distribution (Validation number: REC-16-12-21-02-S). 

*Data collection process*

Before data collection, the authors considered practical issues that may affect the precision of the study. These issues could be withdrawal, failure, or refusal to give valid responses to particular items in the questionnaire. Henceforth, to avoid any degree of attrition and achieve a desired sample size, 525 residents were approached in the city of Sharjah, a major city in the UAE,in public venues such as parks, residential compounds, and shopping malls, via convenience sampling. An information sheet was provided to the participants indicating the significance of the study and its objectives. Participants were approached based on availability and willingness to participate in the study. Inclusion criteria were UAE residents (both locals and expatriates), aged 18 years or more who can communicate in Arabic or English. Participants who did not match these criteria were excluded. Induction training was provided to all researchers regarding data collection and a standardization process was implemented to ensure answers to all enquiries are calibrated. This included obtaining written informed consent, then handing the questionnaire and giving the participants time and privacy to answer questions. Data collectors were available to answer any inquiries and clarify any ambiguities. If the participants had problems understanding one of the questions, a standard explanation was provided by the researchers to avoid leading and biased questions. Confidentiality was maintained as the collected data was only available to the researchers. 

*Statistical analysis*

Data was entered and analyzed using the Statistical Package for Social Sciences (SPSS) Version 21 software (IBM Corp., Armonk, NY, USA). Scores were generated for knowledge, attitudes, and practices to allow for easier comparison and correlation. The total knowledge score was calculated by giving one point to every true statement chosen by the participants. Incorrect statements were not awarded. Participants were stratified based on their total scores as excellent (> 66%), average (33 – 66%) and poor (< 33%). The same system was implemented to assess participants’ attitudes and practices. Data was expressed in the form of frequencies and percentages where applicable. A variety of inferential statistical tests were employed to study the relationship between variables including Chi-square for categorical data. ANOVA and Student’s t-tests were conducted to compare mean scores among the different groups. A p-value of ≤ 0.05 was considered to be statistically significant. Quantitative variables were reported as a mean ± standard deviation if the data was in normal distribution. Median values were reported if the data was not in normal distribution. 

## Results

The questionnaire was distributed to 525 participants, 500 questionnaires were completed, yielding a response rate of 95% (females = 58%, males = 42%). The majority, 83.4% of our participants have received or are currently pursuing higher degree education. The rest of the participants have attained only primary or secondary education in school. As for occupation, 9.9% of participants were found to be healthcare professionals. The socio-demographic data of the participants are summarized in Table 1.

*General knowledge regarding H. pylori*

General knowledge about *H. pylori* was generally poor among the target population. Remarkably, only 24.6% have previously heard of *H. pylori*, of which 79.2% were participants from medical backgrounds.

We assessed the general knowledge of the population with regards to stomach ulcers and the bacterium *H. pylori* and its health impacts. While 39% of the population recognized the relationship between *H. pylori* and gastric cancer, 48.8% of our participants were aware of the association between *H. pylori* and gastric ulcers. Only 45% of the participants reported that bacterial infections could lead to the development of a stomach ulcer. Interestingly, only 27.8% recognized the link between the use of medications such as NSAIDs on the development of stomach ulcers and approximately 40% did not recognize the link between poor practices and lifestyle choices and stomach ulcer development. Surprisingly, only 3% of the participants thought that stress could be linked to stomach ulcer development. 

Other common causes of stomach ulcers as reported by our participants are summarized in Table 2. 

The level of knowledge among different sets of groups including gender, educational level and field of work were compared using unpaired t-test, as seen in Table 3. Females had higher average knowledge scores (58.22%) compared to males (56.25%) (p = 0.008*). Compared to participants with a non-medical background (56.74%), those who are in different medical fields (64.08%) had a higher average knowledge score (p < 0.0001*). The educational level did not contribute to the level of knowledge of our participants (p = 0.8271) as reported in the same table. When asked about remedies they would engage in or suggest to others to relieve symptoms of stomach ulcers, 55.8% incorrectly said they would suggest drinking milk which could relieve symptoms of gastroesophageal reflux or heartburn but not symptoms of stomach ulcers. Similarly, 34.8% would suggest using an antacid for relief of stomach ulcer symptoms, a drug commonly used to relieve symptoms of acid reflux. Other remedies our participants reported are summarized in Table 4.

Knowledge about routes of transmission of stomach bacteria such as *H. pylori* was also relatively poor. Few recognized low socioeconomic conditions (23.2%) and overcrowding, sharing utensils and toothbrushes (26.6%), contaminated water sources and food (46.2%) as possible routes of transmission. 

We evaluated the knowledge of the participants regarding *H. pylori* and its possible health impacts. Participants were asked to point out diseases that could develop as a result of *H. pylori* infection: 49.3% recognized the link between stomach ulcers and *H. pylori*; 45.5% recognized the link between duodenal ulcers and *H. pylori*, only 8.3% suggested that dyspepsia could develop as a result of *H. pylori* infection. Renal failure, a common health effect of *H. pylori* was only reported by 9.7% of our population. Alarmingly, only 39.4% of the participants recognized gastric cancer as a sequelae of *H. pylori* induced gastric ulcer. 

Family and friends (34.7%) and place of study (28.1%) were the most frequently reported sources of information about *H. pylori* induced gastric ulcer. Rest of the resources could be seen in Table 5.

*Attitudes towards H. pylori*

Participants’ attitudes regarding *H. pylori* infection were suboptimal, only 33% of the population said they would seek medical help or suggest it to others in the event of suffering from symptoms suggestive of stomach ulcers. However, 76.2% claimed they would undergo an endoscopy to further assess stomach ulcers in case they were diagnosed with *H. pylori* despite the discomfort associated with it. General attitudes of the participants towards preventing acquiring *H. pylori* are summarized in Table 6. 

Attitudes regarding the prevention of *H. pylori* and stomach ulcers, reported in Table 7 using ANOVA test, were significantly better in patients who suffered from ALARM symptoms in comparison to those who did not (F (2,497) = 2.685, p = 0.0208*). 

*Practices and daily habits*

Approximately 84% of our participants showed an overall average to excellent practices. The most frequent practices the population engages in on a regular basis were drinking coffee (48.4%), eating spicy food (31.2%), drinking soft drinks (16.8%), smoking (28.8%), and using anti-inflammatory drugs (11.2%).

We compared practices among different groups including gender, educational level and field of work using unpaired t-test, reported in Table 8. Females were found to have higher mean practice scores (81.28%) in comparison to males (72.72%) (p < 0.0001*). The educational level and the field of work were not associated with better practice scores (p = 0.2377, p = 0.8582 respectively).

**Table 1:** Demographic Data of Participants in the Study

Demographic variable	Frequency	Respondents’ (%)
Gender		
Male	210	42.10%
Female	289	57.90%
Age		
18 - 29	185	37.40%
30 - 39	174	35.20%
40+	136	27.50%
Nationality		
Emirati	58	12.00%
Arab	290	60.20%
Non-Arab	134	27.80%
Educational level		
Elementary school	7	1.50%
High school	73	15.10%
University student	134	27.80%
University graduate	268	55.60%
Emirate		
Abu Dhabi	21	4.20%
Dubai	70	14.00%
Sharjah	379	75.80%
Rest of Northern Emirates	30	6.00%
Field of work		
Medical	48	9.90%
Non-Medical	438	90.10%

**Table 2:** Responses about Possible Causes of Gastric Ulcers

Remedies	Frequency	Respondents’%
Drinking milk	221	44.20%
Using an antacid	174	34.80%
Drinking water	241	48.20%
Using anti-inflammatory medications	33	6.60%
Drinking 7-up	63	12.60%
Sleeping on the stomach	47	9.40%
Seeking medical help	165	33%
Herbal drinks	174	34.80%
Eating	57	11.40%
Ignoring it	50	10%

**Table 3:** Knowledge Scores among Ddifferent Groups Including Gender, Educational Level, and Field of Work

Demographic variable	Frequency	Respondents’ (%)	Knowledge Score (%)	Test value	p-value
Gender					
Male	210	42.10%	56.25%	2.901*	0.008
Female	289	57.90%	58.22%		
Educational level					
School Graduates	80	16.60%	56.91%	0.7190*	0.8271
Higher Education	402	83.40%	57.98%		
Field of work					
Medical	48	9.90%	64.08%	6.695*	<0.0001
Non-Medical	438	90.10%	56.74%		

*Test is unpaired t-test.

**Table 4:** Remedies Recommended or Used by Participants to Alleviate Symptoms of Gastric Ulcers

Remedies	Frequency	Respondents’%
Drinking milk	221	44.20%
Using an antacid	174	34.80%
Drinking water	241	48.20%
Using anti-inflammatory medications	33	6.60%
Drinking 7-up	63	12.60%
Sleeping on the stomach	47	9.40%
Seeking medical help	165	33%
Herbal drinks	174	34.80%
Eating	57	11.40%
Ignoring it	50	10%

**Table 5:** Sources of Knowledge towards *H. pylori,* Its Routes of Transmission, Prevention, and Associated Diseases

Source of knowledge	Frequency	Respondents’%
Personal reading	103	20.70%
Social media	74	14.90%
Mass media (News, TV, and radio)	41	8.30%

**Table 6:** General Attitudes of Participants towards Prevention of *H. pylori* Infection

Attitudes	Frequency	Percentage (%)
Ensure clean water and food consumption	378	75.60%
Improve self-hygiene	311	62.20%
Getting frequent checkups	305	61%
Avoid sharing utensils	249	49.80%
None of the above	11	2.20%

**Table 7:** Effect of the Severity of Symptoms Experienced by the Participants on Their Positive Attitudes towards Prevention, Early Detection, and Management of *H. pylori*

Severity of Symptoms	Attitude Score (%)	Test value	p-value
Mild	53.23%	2.685**	0.0208
Moderate	53.38%		
Severe	57.63%		

**Test is ANOVA test

**Table 8:** Practice Scores among Different Groups, Including Gender, Educational Level, and Field of Work

Demographic variable	Frequency	Respondents’ (%)	Practice Score (%)	Test value	p-value
Gender					
Male	210	42.10%	72.72%	4.876*	<0.0001
Female	289	57.90%	81.28%		
Educational level					
School Graduates	80	16.60%	75.11%	1.18*	0.2377
Higher Education	402	83.40%	78%		
Field of work					
Medical	48	9.90%	78.08%	0.1787*	0.8582
Non-Medical	438	90.10%	77.55%		

## Discussion

To our knowledge, our study is the first of its kind to survey public KAPs with regards to *H. pylori* in the UAE. The limited awareness of *H. pylori* infection is alarming, given that the risk of *H. pylori*-induced gastric cancer can be reduced with early screening in high-risk populations and a simple course of antibiotics. Our study aimed to assess respondents’ knowledge of gastric ulcers, their causes – specifically regarding *H. pylori* –, possible remedies and complications. 

General knowledge regarding *H. pylori *General knowledge of the Sharjah population with regards to *H. pylori* gastric ulcers is poor. Knowledge about the common causes of gastric ulcers has also been found poor; while nearly half of the respondents recognized bacterial infections as a cause of gastric ulcers, only a quarter have previously heard of *H. pylori*. This is consistent with a study conducted in China where 22% and 35% of the seropositive and seronegative patients reported hearing about *H. pylori*, respectively (Xia et al., 2012). As reported in our results section, more participants (48.8%) were aware of the association between *H. pylori* and gastric ulcers than those who recognized the relationship between *H. pylori* and gastric cancer (39%), which shows a clear lack of knowledge of the attributable effects and the pathophysiology of this microorganism on the gastric mucosa, leading to gastric cancer. While curling ulcer is a common surgical entity defined as a gastric erosion due to reduced plasma volume following burns, only 3.6% recognize skin burns and major bleeding as a cause of stomach ulcers. Moreover, only 3% of our respondents were aware that psychological stress can increase the risk of developing stomach ulcers. This is inconsistent with other studies in which stress was considered by the participants to be the highest risk factor for developing not only stomach ulcers (60.0%) (CDC, 1997), but also gastric cancer (73.5%) (Oh et al., 2009).

In our study, less than half of the participants recognized contaminated food and water as a means of transmission of the bacterium. This is consistent with other studies where participants had been asked how *H. pylori* infection can be acquired and only 24% correctly answered water and poor food preparations as the main sources (Chen et al., 2005). Another study has reported oral transmission with 12.6% when participants were asked about transmission (shin et al., 2013). This emphasizes the importance of water sanitation and hygienic practices such as handwashing, which should be promoted by the local health authorities to combat pathogenic infections caused by waterborne microorganisms (Wynne et al., 2013).

As expected, respondents with medical backgrounds were found to have more knowledge about *H. pylori* and its attributable effects than those from other non-medical backgrounds (p <0.0001*).

Discrepancy between males and females with regards to their knowledge and practices was significant, with females having better knowledge and practice scores. Females were found to have a higher average knowledge score (p = 0.008*) and engaged in practices that predisposed them to *H. pylori* much less frequently that their male counterparts (p <0.0001*).

*Attitudes towards H. pylori*

Patients were inquired on whether they suffer from symptoms of anemia, loss of weight, anorexia, dysphagia, odynophagia, melena, and hematemesis - also known as ALARM symptoms. These symptoms are commonly attributed to complicated gastric ulcer disease. Participants who suffered from ALARM symptoms were significantly better with regards to screening, avoidance, and management than those who suffer from mild or moderate symptoms (p = 0.0208*). This could be justified by the discomfort associated with peptic ulcers leading to better attitudes to avoid suffering from these symptoms in the future and avoid risk of development of gastric ulcers or infection with *H. pylori*. 

While 90% of subjects reported they would not ignore symptoms of stomach ulcers, only 33% stated they would seek medical help. Moreover, 39% of respondents indicated they would not be willing to undergo frequent medical check-ups or screening to avoid infection with *H. pylori*. This could be due to their preference for non-medical alternatives or herbal remedies, as is common in this part of the world, or concerns regarding medical costs for uninsured subjects. It is also likely that many patients assume their symptoms will improve eventually without the need for medical intervention. 

To confirm a diagnosis of *H. pylori* infection, a confirmatory test such as biopsy urease testing via endoscopy, urea breath test or stool antigen test is recommended (Lehours, 2018) Endoscopy has a high sensitivity and specificity of 90% and 95%, respectively (Malfertheiner et al., 2016). It is indicated in patients who suffer from upper gastrointestinal symptoms. Factors considered when choosing a confirmatory test include prevalence, cost, recent use of medications and test availability (Wang, 2015). Despite the associated discomfort, 76% of our participants said they would agree to undergo an endoscopy to diagnose *H. pylori*-associated stomach ulcers if necessary. 

*Practices and daily habits*

Majority of our participants showed overall excellent practices (84%). Good practices have been registered in another study in 74.5% of the population studied (Abongwa et al., 2017). Most participants (71.4%) were found to engage in at least one of the practices listed that increase the risk of developing gastric ulcers such as smoking, drinking coffee regularly or using non-steroidal anti-inflammatory drugs such as Aspirin or Ibuprofen. These practices not only predispose them to developing gastric ulcers but also increase the risk of future progression to gastric cancer. This was especially seen with coffee consumption and cigarette smoking. Interestingly, recent studies that analyzed the relation between cigarette smoking and *H. pylori* risk produced controversial results. While several studies found that the risk of *H. pylori* infection rose drastically with smoking (A-Ameri and Alkadasi, 2013; Ozden et al., 2004), one study suggested that it decreases the risk (Kanbay et al., 2005). Other studies revealed no association between smoking and *H. pylori* infection (Constanza et al., 2004; Woodward, 2000). A study in Germany suggested a dose-dependent relationship of coffee consumption to active infection in that it can increase the risk of seroconversion around 5 times among those who drink more than 2 cups of caffeinated drinks per day (Brenner et al., 1999). 

Handwashing before preparing meals is generally thought to be a good practice to reduce getting infections (Amaral et al., 2017). Unexpectedly however, Abebaw et. al linked a higher prevalence of *H. pylori* with hand washing before preparing meals (Abebaw et al., 2014). This is inconsistent with a study conducted by Lee et al. which showed no increased prevalence of *H. pylori* with handwashing (Lee et al., 2012). In our study, 62.2% of respondents stated they would be willing to improve their self-hygienic practices to avoid being infected with *H. pylori*. This elucidates the need for more studies to delineate the link between *H. pylori* and handwashing to formulate future guidelines with regards to the matter at hand. 

The difference between respondents from a medical background in comparison to those from non-medical backgrounds was not significant with regards to their practices (p = 0.8582). It was expected that due to practicing in the medical field, those participants would have more awareness regarding the effect of negative practices on the future development of gastric ulcers.

*Future directions*

We would also like to highlight the importance of a well-balanced nutritional diet and caloric distribution in symptomatic peptic ulcer patients; thus, additional studies which address this issue are necessary (Vomero and Colpo, 2014).

*H. pylori* antibiotic resistance is the main factor affecting efficacy of current therapeutic regimens, and the best alternative would be a prophylactic vaccine to potentially suppress the emergence of new cases of *H. pylori*-induced gastric ulcers (Abadi, 2016). There is no currently available vaccine against *H. pylori*. However, a vaccine known as IMX101 developed by Imevax (Sutton and Boag, 2019) has recently completed phase one trials. While more ambitious solutions such as vaccines could theoretically eliminate *H. pylori* infections, simpler approaches encompassing increasing awareness and education would go a long way in reducing disease burden and facilitating early detection and intervention programs.

*Limitations*

Our research used a convenience sampling method, which might have affected the generalizability of the results. This was manifested by our sample being confined to Sharjah. Not to mention, the relationship between health-related behavior and the characteristics measured by KAP surveys are complex and difficult to quantify. Furthermore, translation of the questionnaire was from English to Arabic and not the other way around. The questionnaire used was developed by the authors; a validated questionnaire was not used in this study. In order to confirm validity and reliability of the scale, further research is required to analyze the psychometric property of this questionnaire. In the future, it would be interesting to assess the self-risk of contracting *H. pylori* infection in the population.

In conclusion, our data demonstrated clear evidence about the lack of knowledge of the general population with regards to gastric ulcers generally and *H. pylori*-induced gastric ulcers, specifically. As such, it is imperative to implement educational outreach programs with the aim to increase public awareness about *H. pylori*, its routes of transmission and its health impacts. More importantly, screening and surveillance for early detection and intervention would be of great use in reducing the incidence of *H. pylori*-induced gastric ulcer and its complications such as gastric cancer. These could be easily avoided by ensuring early management with simple antibiotics. 

## Author Contribution Statement

A.M., M.A., L.O., H.B. contributed to data collection, data analysis, literature review and manuscript writing and editing. A.A. contributed to data collection, data analysis and literature review. M.F. contributed to data collection and literature review. 
